# The relationship between T-lymphocyte subset infiltration and survival in patients with prostate cancer

**DOI:** 10.1038/sj.bjc.6601943

**Published:** 2004-07-13

**Authors:** P A McArdle, K Canna, D C McMillan, A-M McNicol, R Campbell, M A Underwood

**Affiliations:** 1 University Department of Surgery, Royal Infirmary, Glasgow G31 2ER, UK; 2 University Department of Pathology, Royal Infirmary, Glasgow G31 2ER, UK; 3 University Department of Urology, Royal Infirmary, Glasgow G31 2ER, UK

**Keywords:** CD4+, CD8+, T-lymphocytes, survival, prostate cancer

## Abstract

The relationship between tumour stage, T-lymphocyte subset infiltration and survival was examined in patients with prostate cancer (*n*=80). On multivariate analysis PSA (HR 2.47, 95% CI 1.27–4.83, *P*=0.008) and CD4+ T-lymphocyte count (HR 2.29, 95% CI 1.25–4.22, *P*=0.008) had independent significance. Increased CD4+ T-lymphocyte infiltration within the tumour was stage independent and associated with poor outcome in patients with prostate cancer.

It has long been recognised that disease progression in cancer patients is not solely determined by the characteristics of the tumour but also by the host response. Indeed, there is increasing evidence that both local and systemic inflammatory responses play an important role in the progression of a variety of common solid tumours (Coussens and Werb, 2002).

For example, in patients with colorectal cancer, there is good evidence that the presence of a pronounced lymphocytic infiltration within the tumour is associated with improved survival (Jass *et al*, 1987; Ropponen *et al*, 1997, Nielsen *et al*, 1999). More recently, the ability to identify lymphocyte subsets has led to renewed interest in the relationship between the tumour inflammatory infiltrate and outcome. Indeed, increased infiltration of the tumour by CD8+ and CD4+ T-lymphocytes has been shown to be associated with increased survival in patients with colorectal cancer (Naito *et al*, 1998; Ali *et al*, 2004). In contrast, the presence of an increased infiltration by CD4+ or CD8+ T-lymphocytes has been associated with decreased survival in patients with renal cancer (Nakano *et al*, 2001, Bromwich *et al*, 2003).

The relationship between lymphocytic infiltration and survival in other urological cancers, for example prostate cancer, is less clear. Vesalainen *et al* (1994) reported that, in a cohort in which approximately 30% of patients had metastatic disease, tumours with a dense tumour lymphocyte infiltration were associated with higher survival rates than tumours with absent or decreased infiltrates. In contrast, Irani *et al* (1999) reported that, in patients undergoing radical prostectomy, an increased inflammatory cell infiltrate within the tumour was associated with an increased risk of tumour recurrence.

To date, the relationship between lymphocyte subset infiltration and survival does not appear to have been assessed in patients with prostate cancer. The aim of the present study was to examine the relationship between CD4+ and CD8+ T-lymphocyte infiltration and survival in patients with prostate cancer.

## PATIENTS AND METHODS

### Patients

Patients who underwent radical prostatectomy for histologically proven prostate cancer (*n*=11) or who were diagnosed as having prostate cancer following transurethral resection of prostate for bladder outflow obstruction (*n*=69) were included in the study. Clinical details recorded included age, stage as described by Wilkinson and Hamdy (2001), tumour grade as defined by Chodak *et al* (1994), circulating concentrations of PSA and haemoglobin at diagnosis, and subsequent treatment.

All patients were followed up in the Department of Urology. The date and cause of death was obtained from the cancer registry.

The study was approved by the local ethics committee.

### Methods

#### Immunohistochemistry

Blocks from the primary tumour were fixed in 10% buffered formalin and embedded in paraffin wax. One representative block of tumour was selected from each patient. Sections (4 *μ*m) were cut and mounted on slides coated with aminopropyltriethoxysilane. Sections were then immunostained using the peroxidase-based Envision (Dako, Cambridgeshire, UK) technique as previously described (Bromwich *et al*, 2003). The primary antibody for CD4 was mouse monoclonal (Vector, Peterborough, UK) and that for CD8 was mouse monoclonal (Dako, Cambridgeshire, UK).

#### Morphometry

Quantitative analysis of the lymphoid infiltrate was performed using point counting (Anderson and Dunnill, 1965) with a random sampling technique. With this method, the volume occupied by any given component (volume density) is expressed as a percentage of the total volume of the tissue. A 100-point ocular grid was used at × 400 magnification and 30 fields were counted per case for CD4+ and CD8+ immunopositive cells.

CD4+ and CD8+ T-cells within the tumour (including both the cancer cell nests and surrounding stroma) were counted. Any normal tissue on the slide was excluded from the analysis.

This final method was designed on the basis of a pilot study, which demonstrated that the volume density of CD4+ and CD8+ of two observers reached a plateau after 25–30 fields. This pilot study also demonstrated that CD4+ and CD8+ counts were equivalent to the CD3+ counts (unpublished data). The observers (McArdle and Canna) were blinded to the clinical outcome of the patient.

### Statistics

Data are presented as median and range. Where appropriate, comparison of patient groups was carried out using contingency table analysis (*X*^2^) and the Kruskal–Wallis test for analysis of variance. For the purpose of survival analysis T-lymphocyte subset counts were grouped into tertiles as previously described (Nielsen *et al*, 1999). Survival analysis was performed using the Cox proportional hazard model. Deaths up to August 2003 have been included in the analysis. Analysis was performed using SPSS software (SPSS Inc., Chicago, IL, USA).

## RESULTS

In total, 80 patients were included in the study. The baseline characteristics, according to stage, are shown in Table 1

**Table 1:** Baseline characteristics, according to stage, of patients with prostate cancer

	**Localised (*n*=20)**	**Locally advanced (*n*=37)**	**Metastatic (*n*=23)**	***P*-value**
Age group (⩽70/>70)	11/9	22/15	5/18	0.013
Tumour grade (low/intermediate/high)	5/9/5	10/16/11	3/10/10	0.635
PSA (<4/4–10/11–100/>100 ng ml^−1^)	7/5/7/0	4/6/22/3	0/1/12/10	<0.001
Haemoglobin (⩾12/<12 g l^−1^)	18/0	28/7	15/7	0.035
% *tumour volume*				
CD4+^a^	0.42 (0.03–1.70)	0.40 (0.07–1.90)	0.53 (0.07–1.63)	0.475
CD8+^a^	0.87 (0.27–1.70)	0.73 (0.27–3.00)	0.83 (0.27–1.90)	0.882
CD4+ plus CD8+^a^	1.30 (0.30–3.13)	1.17 (0.33–4.90)	1.23 (0.50–3.20)	0.801
				
Treatment (radical local/androgen deprivation)	11/9	9/28	1/22	0.001
Alive/dead (cancer specific/intercurrent)	11/9	19/18	9/14	0.018
	2/7	8/10	12/2	

a Median (range).

. Patients with metastatic disease were older, had higher circulating concentrations of PSA and had lower haemoglobin concentrations. A total of 21 patients underwent radical local therapy (prostatectomy or radical radiotherapy) as primary treatment. The remaining 59 patients received androgen deprivation therapy. The median (range) for percentage volume of CD4+ T-lymphocytes was 0.40 (0.03–1.90). The median (range) for percentage volume of CD8+ T-lymphocytes was 0.78 (0.27–3.00).

The minimum follow-up was 26 months; the median follow-up of survivors was 71 months. In all, 41 patients died during follow-up, 22 of prostate cancer and 19 of intercurrent disease. The mean cancer specific-survival for those with localised, locally advanced and metastatic disease was 120, 98 and 66 months respectively (*P*=0.001).

On univariate analysis, advanced stage (*P*<0.01), elevated PSA concentrations (*P*<0.01), decreased haemoglobin (*P*<0.05) and increased CD4+ T-lymphocyte counts (*P*<0.05) were associated with reduced cancer specific survival (Table 2

**Table 2:** Clinicopathological characteristics in patients with prostate cancer: univariate survival analysis

	**Patients (*n*=80)**	**Hazard ratio (95% CI)**	***P*-value**
Age group (⩽70/>70)	38/42	2.38 (0.97–5.85)	0.059
Stage (localised/locally advanced/metastatic)	20/37/23	2.99 (1.52–5.88)	0.002
Tumour grade (low/intermediate/high)	18/36/26	1.41 (0.80–2.51)	0.238
PSA (<4/4–10/11–100/>100 ng ml^−1^)	11/12/41/13	2.50 (1.38–4.52)	0.002
Haemoglobin (⩾12/<12 g l^−1^)	61/14	3.38 (1.28–8.90)	0.014
*Tumour counts*			
CD4+ T-lymphocyte count (tertiles)	26/28/26	2.03 (1.15–3.59)	0.015
CD8+ T-lymphocyte count (tertiles)	26/28/26	1.46 (0.85–2.52)	0.170
CD4+ plus CD8+ count (tertiles)	26/28/26	1.69 (0.97–2.97)	0.065
			
Treatment (radical local/androgen deprivation)	21/59	2.08 (0.70–6.17)	0.188

). On multivariate analysis PSA (HR 2.47, 95% CI 1.27–4.83, *P*=0.008), haemoglobin (HR 3.48, 95% CI 1.30–9.30, *P*=0.013) and CD4+ T-lymphocyte count (HR 2.29, 95% CI 1.25–4.22, *P*=0.008) retained independent significance. There was no significant correlation between PSA concentrations and either CD4+ (*P*=0.539) or CD8+ (*P*=0.202) T-lymphocytes. There was also no significant correlation between Gleason scores and either CD4+ (*P*=0.246) or CD8+ (*P*=0.409) T-lymphocyte counts.

When those patients with localised or locally advanced prostate cancer (*n*=57) were examined in univariate analysis, only CD4+ T-lymphocyte count achieved statistical significance (HR 2.88, 95% CI 1.15–7.22, *P*=0.024).

## DISCUSSION

The results of the present study show that the presence of an increase in CD4+ T-lymphocyte infiltrate was associated with poor cancer specific survival, independent of stage, in patients with prostate cancer. Furthermore, when the analysis was confined to those patients with localised or locally advanced disease, only CD4+ T-lymphocyte count predicted survival. These results are consistent with those of Irani *et al* (1999) but not with those of Vesalainen *et al* (1994).

The reasons for the difference in the results of the present study and those of Vesalainen *et al* (1994) are not clear. Both cohorts included a wide spectrum of disease and both had a similar proportion of patients with metastatic disease. However, the apparent discrepancies may reflect differences in methodology, including the way in which the inflammatory infiltrate was assessed. Vesalainen and his co-workers assessed the density of tumour infiltrating lymphocytes on simple staining with haematoxylin and eosin. Moreover, the type of lymphocyte was not assessed. In contrast, in the present study, T-lymphocyte subsets were identified by immunohistochemistry and the density was assessed using a point counting technique. This approach provided a more objective assessment and circumvented the problem of variation in distribution of lymphocytes within an individual tumour.

Why the presence of an increase in CD4+ T-lymphocyte infiltrate would be associated with poor cancer specific survival in patients with prostate cancer is unclear since it would seem logical that an increased T-lymphocyte infiltrate would help control and even destroy tumour cells. However, it has been suggested that many of the T-lymphocytes found in tumour beds are inactive and therefore do not contribute to effective antitumoral immunity. Indeed, this concept is consistent with the observation that downregulation of MHC I expression may help tumours cells escape immune surveillance (Naoe *et al*, 2002).

In summary, the results of the present study have shown that the presence of increased CD4+ T-lymphocyte infiltration within the tumour was associated with poor outcome, independent of stage, in patients with prostate cancer.
